# Impairment of *IGF2* gene expression in prostate cancer is triggered by epigenetic dysregulation of *IGF2*-DMR0 and its interaction with KLF4

**DOI:** 10.1186/s12964-017-0197-7

**Published:** 2017-10-10

**Authors:** Undraga Schagdarsurengin, Angela Lammert, Natalie Schunk, Diana Sheridan, Stefan Gattenloehner, Klaus Steger, Florian Wagenlehner, Temuujin Dansranjavin

**Affiliations:** 1 Clinic of Urology, Pediatric Urology and Andrology, Justus-Liebig-University Giessen, Rudolf-Buchheim-Str. 7, 35392 Giessen, Germany; 2 Epigenetics of Urogenital System, Justus-Liebig-University Giessen, Schubertstr. 81, 35392 Giessen, Germany; 3 Department of Signal Transduction of Cellular Motility, Internal Medicine V, Justus-Liebig-University Giessen, Aulweg 128, 35392 Giessen, Germany; 4 Institute of Pathology, Justus-Liebig-University Giessen, Langhansstr. 10, 35392 Giessen, Germany; 5 Molecular Andrology, Justus-Liebig-University Giessen, Schubertstr. 81, 35392 Giessen, Germany

**Keywords:** IGF2, IGF2-DMR0, KLF4, Transcription regulation, Prostate cancer

## Abstract

**Background:**

Human cancer cells often exhibit impaired *IGF2* expression and the underlying mechanisms are multifaceted and complex. Besides the well-known imprinting control region *IGF2/H19*-ICR, the involvement of a differentially methylated region in the promoter P0 of *IGF2* gene (*IGF2*-DMR0) has been suggested. Here, we evaluate several mechanisms potentially leading to up- and/or down-regulation of *IGF2* expression in prostate cancer and present a novel role of Kruppel-like factor 4 (KLF4) as a transcriptional regulator of *IGF2* binding in *IGF2*-DMR0.

**Methods:**

Putative binding sites for transcription factors were identified in *IGF2*-DMR0 using JASPAR CORE database. Gene expressions were analyzed by RT-qPCR in prostate carcinoma and adjacent benign prostate hyperplasia samples obtained by radical prostatectomy (86 RP-PCa and 47 RP-BPH) and BPH obtained by transurethral prostate resection (13 TUR-BPH). Pyrosequencing and qMSP were used for DNA methylation studies in *IGF2*-DMR0, *IGF2/H19*-ICR and Glutathione-S-transferase-P1 (GSTP1) promoter. Loss of imprinting (LOI) was analyzed by RFLP. Copy number variation (CNV) test was performed using qBiomarker CNV PCR Assay. KLF4-binding and histone-modifications were analyzed by ChIP-qPCR in prostate cancer cell lines exhibiting differentially methylated *IGF2*-DMR0 (LNCaP hypomethylated and DU145 hypermethylated). KLF4 protein was analyzed by western blot. Statistical associations of gene expression to methylation, *IGF2* LOI and CNV were calculated by Mann-Whitney-U-test. Correlations between gene expression and methylation levels were evaluated by Spearman’s-Rank-Correlation-test.

**Results:**

We found a significant reduction of *IGF2* expression in the majority of RP-PCa and RP-BPH in comparison to TUR-BPH. Analyzing potential molecular reasons, we found in RP-PCa and RP-BPH in comparison to TUR-BPH a significant hypomethylation of *IGF2*-DMR0, which coincided with hypermethylation of *GSTP1*-promoter, a prominent marker of prostate tumors. In contrast, *IGF2* LOI and CNV did not associate significantly with up- and/or down-regulation of *IGF2* expression in prostate tumors. By analyzing *IGF2*-DMR0, we detected a consensus sequence for KLF4 with a z-score of 7.6. Interestingly, we found that KLF4 binds to hypomethylated (17%) *IGF2*-DMR0 enriched with H3K9me3 and H3K27me3 (LNCaP), but does not bind under hypermethylated (85%) and H3K4me3-enriched conditions (DU145). *KLF4* expression was detected in TUR-BPH as well as in RP-BPH and RP-PCa and showed a highly significant correlation to *IGF2* expression.

**Conclusions:**

Our study demonstrated that in human prostate cancer the impairment of *IGF2* expression is accompanied by hypomethylation of *IGF2*-DMR0. We revealed that KLF4 is a putative transcriptional regulator of *IGF2,* which binds in *IGF2*-DMR0 in dependence of the prevailing epigenetic state in this region. Herewith we provide complementary new insights into *IGF2* dysregulation mechanisms as a critical process in prostate tumorigenesis.

## Background

The insulin-like growth factor 2 (IGF2) is a member of the IGF/insulin signaling pathway and regulates, together with IGF1 and insulin, the cell proliferation and differentiation during embryonic and post-natal development [1–3]. IGF2, which possesses anti-apoptotic as well as mitogenic capacities, is widely expressed lifelong and is involved in regulation of growth [4, 5]. In prostate, IGF2 plays an important role as a paracrine and autocrine regulator of cell growth and was found to be expressed in prostate epithelial cells and in prostate tumor associated stromal cells [6–9].

The expression of the *IGF2* gene is parentally imprinted. In most human cells, *IGF2* expression is restricted to the paternal allele, whereas the maternal allele is repressed [10]. The strict imprinting control is thought to be realized by epigenetic modifications, such as DNA methylation and post-translational histone modifications (PTHMs) at specific loci along the *IGF2* and *H19* gene cluster. The so called imprinting control region (*IGF2/H19*-ICR), which contains a CTCF binding site, is located at the boundary between the *IGF2* and *H19* genes. At normal state, *IGF2/H19*-ICR is methylated in the paternal allele and unmethylated in the maternal allele [10]. In human cancer diseases, IGF2 is often epigenetically dysregulated. Loss of imprinting (LOI) of *IGF2*, aberrant methylation in *IGF2/H19*-ICR and up- or down-regulation of *IGF2* expression, respectively, have been shown to be associated with a number of human tumors including colorectal, breast, liver, bladder, Wilms, ovarian, esophageal, prostate tumors and osteosarcoma [11–21].

In addition to *IGF2/H19*-ICR, it is supposed that a differential methylated region P0 upstream of exon 2 (*IGF2*-DMR0) possesses promoter activities, has a defined methylation status in normal cells (methylated in the paternal and unmethylated in the maternal allele) and a considerable capacity to regulate *IGF2* expression [19, 22, 23]. Numerous studies provide evidence that hypomethylation of the *IGF2*-DMR0 might be also a potent reason for *IGF2* LOI as well as for aberrant *IGF2* expression, and thus, a predictive factor for cancer development [19, 22–26]. In particular, in prostate cancer, it was observed that hypomethylation of *IGF2*-DMR0 correlates with decreased *IGF2* expression [20]. Moreover, a marked *IGF2* LOI was found in adjacent tumor-associated tissues as well as in tumor-free distant regions indicating that IGF2 dysregulation could be an early initiation factor in development of prostate neoplasia [20]. However, the interrelation between methylation changes in *IGF2*-DMR0, LOI and aberrant *IGF2* expression in human and, in particular prostate cancer, is still inconsistent and not clear. The available data were generated in different cells and tumors and are mutually contradictory. Therefore, additional insights with regard to co-factors involved in regulation of *IGF2*-DMR0 and *IGF2* imprinting are needed to elucidate and understand the mechanisms of IGF2 dysregulation during carcinogenesis.

A number of transcription factor families, especially the family of Kruppel-like factors (KLFs), are capable to bind to G/C-rich DNA sequences [27]. One of the members, KLF4, is expressed in a wide variety of tissues including gut, thymus, cardiac myocytes and lymphocytes, and plays an important role in cell proliferation, stem cell self-renewal and maintenance of normal tissue homeostasis [28]. Several studies described and emphasized the role of KLF4 in human cancer and its function in the context of cell and tissue specificity [29–31]. According to previous studies, KLF4 is able to act as a potent tumor suppressor gene in colon and lung carcinogenesis [32, 33] as well as an oncogene that has been shown in breast and skin squamous cell carcinoma [34, 35]. In human prostate, a differential *KLF4* gene expression was observed when comparing normal, hyperplastic and cancerous tissues [36, 37], whereas at protein level KLF4 was detected in a large majority of epithelial prostatic cells, irrespective of malignant transformation [37]. A recent study demonstrated that in prostate cancer cell lines PC3 and LNCaP, KLF4 is associated with the proliferative activity of cells via the KLF4-KRT6/13 pathway [38].

Our current research addressed potential mechanisms leading to *IGF2* dysregulation in prostate carcinogenesis and emphasized the role of KLF4 in regulation of *IGF2*-DMR0. Utilizing prostate cancer cell lines LNCaP and DU145, we demonstrated that KLF4 binds to hypomethylated *IGF2*-DMR0 and affects *IGF2* expression in dependence to prevailing post-translational histone modifications. Analyzing prostate tissues samples from patients with malignant and benign prostate enlargements, we found a highly significant correlation between *IGF2* and *KLF4* expression. Methylation status of *IGF2*-DMR0, but not of *IGF2/H19*-ICR, was associated with PCa and adjacent BPH. Our results reveal that the transcription factor KLF4 is a potent co-factor involved in impairment of *IGF2* expression during prostate carcinogenesis.

## Methods

### Patient samples

Tissue samples from 86 RP-PCa (prostate carcinoma samples obtained by radical prostatectomy; patients’ median age 67, range 52–76), 47 RP-BPH (benign prostate hyperplasia samples adjacent to PCa obtained by radical prostatectomy; patients’ median age 69, range 52–75) and 13 TUR-BPH (benign prostatic hyperplasia samples obtained by transurethral resections of the prostate; patients’ median age 73, range 57–88) were collected at the Department of Urology, Pediatric Urology and Andrology, Justus-Liebig-University (JLU) Giessen, Germany. All patients gave their written informed consent and the study was approved by the ethical committee of the Medical Faculty, JLU Giessen (ethical vote, AZ123/12). Prostate tissue samples were characterized at the Institute of Pathology, JLU Giessen. All clinical and pathological data of analyzed patients are summarized in Table 1. RP-PCa tissue samples with at least 60% of tumor cell amount were selected for this study. Due to technical failures and restricted tumor material not every experiment could be evaluated for the whole number of samples. The numbers of considered samples are given for each experiment in corresponding figures.

**Table 1:** Summary of clinical and pathological parameters of analyzed patients, who underwent radical prostatectomy (RP) or transurethral resection (TUR) of the prostate

Clinical and Pathological Parameters	Specifications	RP-PCa *n* = 86	RP-BPH *n* = 47	TUR-BPH *n* = 13
Age at diagnosis	Median (min-max)	67 (54–76)	69 (52–75)	73 (57–88)
Lymph node metastasis	N0 (absent), n	63	24	n/a
	N1 (present), n	7	3	n/a
	NX (unknown), n	16	20	n/a
Prostate specific antigen (preoperative level in ng/ml)	“<10”, n	17	9	n/a
	“10–15”, n	44	14	n/a
	“>15”, n	25	13	n/a
	Not known, n	–	11	n/a
Pathological status	T2a-T2c, n	43	15	n/a
	T3a-T3b, n	36	11	n/a
	T4, n	7	3	n/a
Gleason score	“≤7”, n	63	32	n/a
	“>7”, n	24	15	n/a

Abbreviations: *RP-PCa* prostate carcinoma obtained by radical prostatectomy, *RP-BPH* benign prostate hyperplasia adjacent to PCa obtained by radical prostatectomy, *TUR-BPH* benign prostate hyperplasia obtained by transurethral resection, *n/a* not applicable

### Cell lines and culture conditions

Human prostate cancer cell lines LNCaP, DU145 and PC3 were obtained from the German Resource Centre for Biological Material (Braunschweig, Germany) and cultured in RPMI-1640 medium (Invitrogen) supplemented with 10% fetal bovine serum (Invitrogen) and 1% Penicillin-Streptomycin solution (Sigma Aldrich). The effect of the DNA methylation inhibitor 5-aza-2′-deoxycytidine (5-aza-CdR, inhibitor of DNA methyltransferase 1) on *IGF2* gene expression was analyzed in LNCaP (17% hypomethylated in *IGF2*-DMR0) and DU145 (85% hypermethylated in *IGF2*-DMR0) cell lines. Therefore, 2 × 10^6^ cells were cultured for 3 days in the presence of 5 μM 5-aza-CdR (Sigma Aldrich). The cells were harvested on the fourth day and utilized for DNA, RNA, protein and chromatin extractions.

### DNA extraction, pyrosequencing and qMSP

**Fig. 1:**
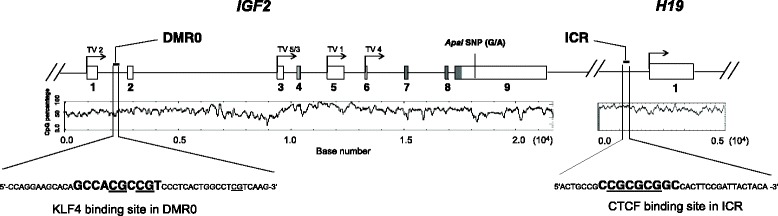
Schematic structure of the human *IGF2/H19* imprinting locus. The *IGF2/H19* gene locus was delineated based on *in-silico* data from NCBI, GenBank (*IGF2*: gene ID 3481, location NC_000011.10; *H19*: gene ID 283120, location NC_000011.10). *IGF2* gene contains nine exons (1–9) and five transcription variants (TV1-TV5). The differential methylated region in the promoter P0 of *IGF2* (DMR0, upstream of exon 2) [19, 50] comprises a putative KLF4 binding site. The KLF4 consensus motif was identified by JASPAR with a z-score of 7.6. The *ApaI* single nucleotide polymorphism (*ApaI* SNP G/A) in *IGF2* exon 9 was used for loss of imprinting studies. The imprinting control region (ICR) upstream of *H19* gene contains a CTCF binding site. The transcription start site and exon 1 of the *H19* gene are indicated. The density of CpGs (CpG percentage) among the *IGF2/H19* imprinting cluster is indicated. The CpGs analyzed by pyrosequencing in the putative KLF4 binding site within *IGF2*-DMR0 and in the CTCF binding site within *IGF2/H19*-ICR are underlined

DNA from tissue samples and cell lines was isolated by standard phenol/chloroform procedure. DNA was precipitated with 1/10 volume NaAc (3 M) and 2.5 volume absolute ethanol. For deamination of unmethylated cytosines, 2 μg DNA were denatured by sodium hydroxide (3 M) and incubated with sodium bisulfite (3.6 M) for 6 h at 56 °C. Bisulfite-treated DNA was purified with Wizard DNA clean-up System (Promega). DNA methylation in *IGF2*-DMR0 and *IGF2/H19*-ICR (Fig. 1) was analyzed by pyrosequencing using primer sets listed in Table 2. Each pyrosequencing procedure included control DNAs (completely methylated and unmethylated EpiTect-control-DNAs (Qiagen). Methylation levels were calculated in percentage by PyroMark Q24 software 2.0 (Qiagen) and depicted in programs. DNA methylation levels show the percentage of cells possessing a complete methylation in an analyzed DNA region. A significant gain or loss of the number of cells possessing a complete methylation in the analyzed DNA region was considered as hyper- and hypomethylation, respectively.

**Table 2:** Primer sets used for pyrosequencing, qMSP, RT-qPCR, ChIP-qPCR and for LOI and CNV studies

Assay	Primer identity	Primer sequence (5′-3′)	PCR-size (bp)
Pyro-sequencing	IGF2-DMR0-PS-F	TTTTTTGTTGTATTTTGGATTTAGATTTTTT	186
	IGF2-DMR0-PS-R	CTCCAAACACCCCCACCTTAA	
	IGF2-DMR0-Seq	GTGGGGAGGGGGTTTATTTTT	
	IGF2-H19-ICR-PS-F	GGGGGCTCTTGCATAGCACATGGGTA	242
	IGF2-H19-ICR-PS-R	ATAAACCCTTATCCTATAAATACCCTCA	
	IGF2-H19-ICR-PS-Seq	GGTATTTTTGGAGGTTTTTTT	
qMSP	GSTP1-MSP-U-F	GATGTTTGGGGTGTAGTGGTTGTT	91
	GSTP1-MSP-U-R	CCACCCCAATACTAAATCACAACA	
	GSTP1-MSP-M-F	TTCGGGGTGTAGCGGTCGTC	97
	GSTP1-MSP-M-R	GCCCCAATACTAAATCACGACG	
RT-qPCR	IGF2-RTPCR-F	GGGCAAGTTCTTCCAATATGA	214
	IGF2-RTPCR-R	TCACTTCCGATTGCTGGC	
	ßActin-RTPCR-F	CGGAGTACTTGCGCTCAGGAGGA	226
	ßActin-RTPCR-R	CCTTCCTTCCTGGGAATGGAGTC	
	KLF4-RTPCR-F	ACTCGCCTTGCTGATTGTCT	127
	KLF4-RTPCR-R	AATTGGCCGAGATCCTTCTT	
	AMACR-RTPCR-F	ACGACTTACAGGACAGCAGA	181
	AMACR-RTPCR-R	CCTTCGTCTTCTCTGCAAAT	
ChIP-qPCR	IGF2-DMR0-ChIP-F	CACCCTGGGGCCAAGGCAGT	100
	IGF2-DMR0-ChIP-R	CTTGAGGGGTCATGGCACGGAAT	
LOI study	IGF2-LOI-ApaI-F	CACCCCCCTCTTTCTCTTCT	245
	IGF2-LOI-ApaI-R	TACTGGGTCCCTCTGACTGCT	
CNV study	ZNF80-GCN-F	CTGTGACCTGCAGCTCATCCT	120
	ZNF80-GCN-R	TAAGTTCTCTGACGTTGACTGATGTG	
	GPR15-GCN-F	GGTCCCTGGTGGCCTTAATT	101
	GPR15-GCN-R	TTGCTGGTAATGGGCACACA	

*Abbreviations*: *qMSP* quantitative methylation specific PCR, *RT-qPCR* reverse transcription of RNA in cDNA followed by quantitative real-time PCR, *ChIP-qPCR* chromatin immunoprecipitation followed by quantitative real-time PCR, *IGF2*-DMR0 differential methylated region in promoter P0 of the insulin-like growth factor 2 gene, *IGF2/H19*-ICR imprinting control region of *IGF2* and *H19* genes, *KLF4* Kruppel-like factor 4, *AMACR* Alpha-methylacyl-CoA racemase, *LOI* loss of imprinting, *CNV* copy number variation, *bp*-base pairs

*GSTP1* promoter methylation was analyzed by quantitative methylation specific PCR (qMSP) using published primer sets [39] for amplification of unmethylated and methylated DNA in *GSTP1* promoter (Table 2). Epi-Tect-control-DNAs (Qiagen) representing 100% methylated and 100% unmethylated DNA were used as calibrators for evaluation of the methylation degree. Methylation independent primers binding in *ß-Actin* gene (PCR product size 133 bp; forward primer: 5′-TGGTGATGGAGGAGGTTTAGTAAGT-3′, revers primer: 5′- AACCAATAAAACCTACTCCTCCCTTAA-3′,) were used as control primers for input DNA. Relative levels of unmethylated (RU) and methylated DNA (RM) in *GSTP1* promoter were calculated using 2^−ΔΔCt^ quantification method. The relative degree of *GSTP1* methylation was calculated in percentage by formula (RM/RU + RM) × 100.

### RNA extraction and RT-qPCR

Total RNA from prostate cancer cell lines and tissue samples was extracted using RNAeasy Mini Kit according to manufacturer’s instruction (Qiagen). The cDNA was generated for each sample using 2 μg RNA and Omniscript Reverse-Transcription (RT) System (Qiagen). Quantitative PCR (qPCR) was performed subsequently using iQ–SYBR-Green-Supermix (BioRad). All RT-qPCR primer sets and PCR product sizes for analyzed genes *IGF2*, *KLF4, AMACR* and *β-Actin* are listed in Table 2. Relative gene expression levels were calculated using 2^−ΔΔCt^ quantitation method by normalization to *β-Actin* (GenEx software, Multid Analyses AB). All PCR amplifications were carried out in triplicates and mean values were calculated.

### Analysis of IGF2 loss of imprinting (LOI)

*IGF2* LOI (bi-allelic expression) was analyzed using a known *IGF2* single nucleotide polymorphism (SNP) G/A in exon 9 [18] (Fig. 1). In order to select heterozygous genotypes, restriction fragment length polymorphism (RFLP) method was performed on DNA samples isolated from prostate tumor tissues using PCR primers shown in Table 2 and the restriction enzyme *ApaI* (recognition site GGGCC^C). The cDNAs (mRNA reverse transcribed in copy DNA) from selected heterozygous samples were generated, and a RFLP analysis was performed using the same PCR primers (Table 2) and enzyme *ApaI*. The bi-allelic expression of *IGF2* was evaluated on cDNA after the separation of *ApaI*-restriction products on 2% agarose gel.

### Analysis of IGF2 copy number variation (CNV)

The CNV of *IGF2* gene was analyzed using the qBiomarker CNV PCR Assay for Human chromosome 11 tile 10,752 (Qiagen). This assay is based on amplification and quantification of an *IGF2* sequence in relation to two reference genes *ZNF80* and *GPR15* [40]. Quantitative PCR reactions were performed according to manufacturer’s protocol in triplicates in 20 μl reaction volume containing Rotor-Gene Sybr Green PCR Master Mix (Qiagen). Data generated by qPCR were analyzed using the commercially available qBaseplus software (Biogazelle NV, Zwijnaarde, Belgium). The reference genes *ZNF80* and *GPR15* were used for normalization of the relative CNV values. Three blood DNA samples from healthy donors, assumed to have normal diploid genomes, were used as additional calibrators for calculation of CNV in different PCR runs. A minimum 2-fold increase of *IGF2* copy numbers in comparison to blood DNA samples was determined as a “gain”, and a decrease of *IGF2* copy numbers (less than 0.5-fold) was determined as a “loss”. Values similar to those in blood DNA samples were considered as “normal”.

### Chromatin immunoprecipitation followed by qPCR (ChIP-qPCR)

LNCaP and DU145 cells (1 × 10^7^ cells) were fixed with 1% formaldehyde for 10 min before quenching with glycine (0.125 M) for 5 min. The nuclei were isolated using nuclear isolation buffer (150 mM NaCl, 10 mM HEPES pH 7.5, 1.5 mM MgCl_2_, 10 mM KCl, 0.5% Nonidet P-40 and 0.5 mM dithiothreitol) and resuspended in nuclear lysis buffer (50 mM Tris-Cl pH 8.1, 10 mM EDTA and 1% SDS). Nucleic lysates were sonicated in order to get DNA fragments ranging from 200 bp to 600 bp. The input DNA controls (10%) were frozen after sonication until further processing. After blocking of unspecific DNA with salmon sperm DNA for 1 hour at 4 °C, the sonicated nuclear material was immunoprecipitated using specific histone antibodies (anti-H3K27me3, −H3K9me3, and –H3K4me3 from Abcam, and anti-KLF4 from R&D Systems) by adding the protein A-conjugated Sepharose (Amersham Biosciences and GE Biosciences). One sample per cell line was incubated without antibodies and used as negative control. After immunoprecipitation over night at 4 °C, protein-DNA complexes bound to Sepharose were washed, and the DNA was isolated according to standard ChIP procedure (Abcam). Enrichment of specific post-translational histone modifications and of KLF4 in *IGF2*-DMR0 was analyzed by qPCR with *IGF2*-DMR0 specific primers (Table 2). Input DNA was used as calibrator.

### Western blot analysis

Whole lysates from LNCaP and DU145 cells (1 × 10^7^ cells) were analyzed for KLF4 expression by western blot. Therefore, cells were washed with ice-cold PBS and scraped on ice after addition of 100 μL RIPA buffer (Sigma Aldrich). Lysed cells were centrifuged at 13.000 rpm for 30 min to precipitate cell debris, and the protein concentrations were measured in the supernatant with Pierce BCA Protein Kit (Thermo Fisher Scientific). Cell lysates (40 μg protein) were mixed with Laemmli buffer containing 10% β-mercaptoethanol, cooked at 100 °C for 5 min and separated in SDS-PAGE. After the protein transfer to a polyvinylidene difluoride membrane, membranes were blocked with 5% BSA and successively incubated with KLF4- (R&D Systems, 1:500) and rabbit IgG HRP-antibody (GeneTex, 1:10,000). Chemiluminescence was measured after addition of Pierce ECL Western Blotting Substrate (Thermo Fisher Scientific).

### Statistical analysis

Non parametric Mann-Whitney U test was used for assessment of statistical differences between the analyzed groups (TUR-BPH, RP-BPH and RP-PCa; LOI and MOI; CNV-gain, −loss and –normal) regarding the methylation levels of *IGF2*-DMR0, *IGF2/H19*-ICR and *GSTP1* promoter as well as for comparison of gene expression levels for *IGF2*, *KLF4* and *AMACR* (normalized to *β-Actin).* Spearman’s rank correlation test was used for correlation of *IGF2*-DMR0 and *IGF2/H19*-ICR- to *GSTP1*-methylation levels and for correlation of *KLF4* to *IGF2* gene expression*.* Data represent median and range (min to max). *P* < 0.05 was considered to be statistically significant and *p* < 0.01 was considered as statistically highly significant.

## Results

### IGF2-DMR0 contains a KLF4 consensus motif

A hypomethylation of the human *IGF2*-DMR0 has been shown to be associated with breast, colorectal and esophageal squamous cell carcinoma [23, 41, 42]. By using the JASPAR CORE database [43], which contained 138 matrices and a subset of TRANSFAC release 10.4 (506 matrices of human and mouse origin), we identified in *IGF2*-DMR0 a putative binding site for the transcription factor KLF4 (Fig. 1). The consensus sequence for KLF4 with the motif 5′-CGGCGTGGC-3′ (complementary sequence 3′-GCCACGCCG-5′) possessed a calculated z-score of 7.6 and is located within a region exhibiting a high CpG-density (Fig. 1).

### Prostate cancer associates with decreased IGF2 expression and hypomethylation of IGF2-DMR0

**Fig. 2:**
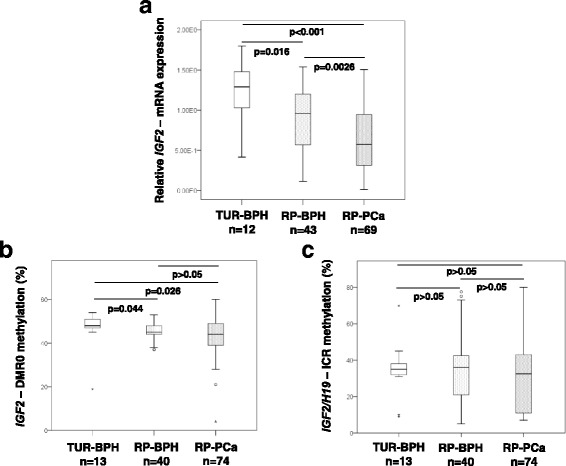
Expression of *IGF2*-mRNA as a function of DNA methylation in *IGF2*-DMR0 and *IGF2/H19*-ICR. **a** *IGF2*-mRNA expression was measured by RT-qPCR in TUR-BPH (benign prostate hyperplasia obtained by transurethral resection of the prostate), RP-BPH (BPH adjacent to prostate carcinoma, PCa, obtained by radical prostatectomy) and RP-PCa samples (PCa obtained by radical prostatectomy). A significantly reduced *IGF2*-mRNA expression was detected among RP-BPH and RP-PCa; **b** DNA methylation in *IGF2*-DMR0 was measured by pyrosequencing in TUR-BPH, RP-BPH and RP-PCa. A significant hypomethylation of *IGF2*-DMR0 was observed particularly in RP-PCa samples in comparison to TUR-BPH. In RP-PCa samples the highest variance of *IGF2*-DMR0 methylation was observed; **c** The methylation levels of *IGF2/H19*-ICR were not significantly different among the three analyzed groups. A relative high deviation of *IGF2/H19*-ICR methylation was found in RP-BPH and RP-PCa samples. *P*-values were determined by Mann-Whitney U test

Tissue samples obtained from TUR-BPH (*n* = 12), RP-BPH (*n* = 43) and RP-PCa (*n* = 69) were analyzed and compared with regard to *IGF2*-mRNA expression. The TUR-BPH samples showed the highest *IGF2* expression and were followed by RP-BPH and RP-PCa (TUR-BPH vs. RP-BPH: *p* = 0.016; RP-BPH vs. RP-PCa: *p* = 0.0026, Mann-Whitney U test) (Fig. 2a). DNA methylation analyses in *IGF2*-DMR0 revealed that RP-BPH and RP-PCa samples are significantly hypomethylated when comparing to TUR-BPH samples (TUR-BPH vs. RP-BPH: *p* = 0.044; TUR-BPH vs. RP-PCa: *p* = 0.026, Mann-Whitney U test). Noticeably, the RP-PCa samples displayed the highest variance of *IGF2*-DMR0 methylation in both directions (hypo- and hypermethylation; median 44%, range 4–60%) (Fig. 2b). In contrast, the methylation *of IGF2/H19*-ICR (imprinting control region with a CTCF binding site located between *IGF2* and *H19* genes, Fig. 1) was not significantly different between the prostate cancer groups TUR-BPH, RP-BPH and RP-PCa (Fig. 2c). The highest variance of *IGF2/H19*-ICR methylation was found in RP-BPH (median 36%, range 5–78%) and RP-PCa samples (median 32.5%, range 7–80%) (Fig. 2c).

### IGF2 LOI in prostate cancer associates with hypermethylation of IGF2/H19-ICR, but not with methylation changes in IGF2-DMR0

**Fig. 3:**
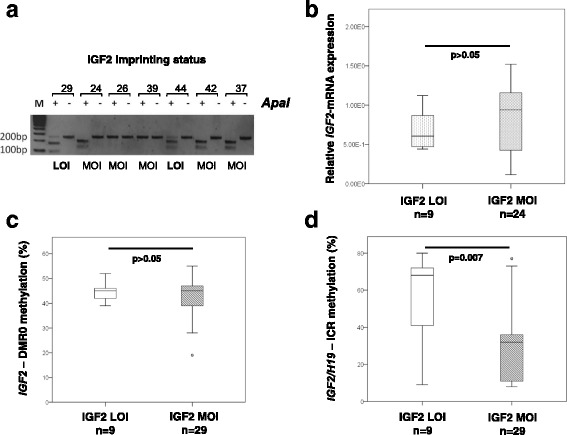
Association of *IGF2* LOI to *IGF2* gene expression and DNA methylation in *IGF2*-DMR0 and *IGF2/H19*-ICR. **a** *IGF2* imprinting status (one or two allele expression) was analyzed by restriction fragment length polymorphism (RFLP, *ApaI*, GGGCC^C) on cDNA generated from tissue samples with heterozygous genotypes. A representative RFLP agarose gel image shows two cases with LOI (loss of imprinting; samples 29 and 44 show bi-allelic *IGF2* expression) and five cases with MOI (maintenance of imprinting; samples 24, 26, 39, 42 and 37 show one-allelic *IGF2* expression); **b** No significant difference regarding *IGF2*-mRNA expression was found between prostate tissue samples exhibiting LOI and MOI; **c** Methylation of *IGF2*-DMR0 was not significantly changed in samples exhibiting LOI in comparison to those with MOI; **d** A significantly higher methylation in *IGF2/H19*-ICR was found in LOI-group. P-values were determined by Mann-Whitney U test

Loss of imprinting (LOI) of *IGF2* is considered to be an epigenetic marker for the risk of human cancer, particularly colorectal cancer [13, 44, 45] and has a potential to affect the gene expression. Although contradictory, some publications also showed an interrelation between *IGF2* LOI and methylation changes in *IGF2/H19* locus [22, 24]. Here, we aimed to address these issues in prostate cancer. Therefore, we analyzed on cDNAs of 38 selected prostate cancer tissue samples with proven heterozygosity (6 TUR-BPH, 13 RP-BPH and 19 RP-PCa) the imprinting status of *IGF2,* i.e. one or two allele expression. The LOI analysis was done by RFLP utilizing the SNP (G/A, *ApaI* recognition site GGGCC^C) in exon 9 of *IGF2* gene (Fig. 1). Among all analyzed G/A heterozygous samples we could identify 9 (1 TUR-BPH, 3 RP-BPH and 5 RP-PCa) with LOI, i.e. bi-allelic expression (Fig. 3a). The remaining 29 samples showed maintenance of imprinting (MOI). Samples with LOI were then compared to those with MOI regarding the *IGF2* expression, and no significant difference was found (*p* > 0.05, Mann Whithney U test) (Fig. 3b). We then compared samples with LOI and MOI with regard to DNA methylation in *IGF2*-DMR0 and in *IGF2/H19*-ICR. Samples with LOI showed a highly significant increase of *IGF2/H19*-ICR methylation (median 68%, range 9–80%) in comparison to samples with MOI (median 32%, range 8.77%) (LOI vs. MOI: *p* = 0.007, Mann-Whitney U test) (Fig. 3d). In contrast, no significant difference between LOI and MOI samples was found regarding the methylation in *IGF2*-DMR0 (Fig. 3c).

### GSTP1 hypermethylation and IGF2-DMR0 hypomethylation are highly correlated in prostate carcinogenesis

**Fig. 4:**
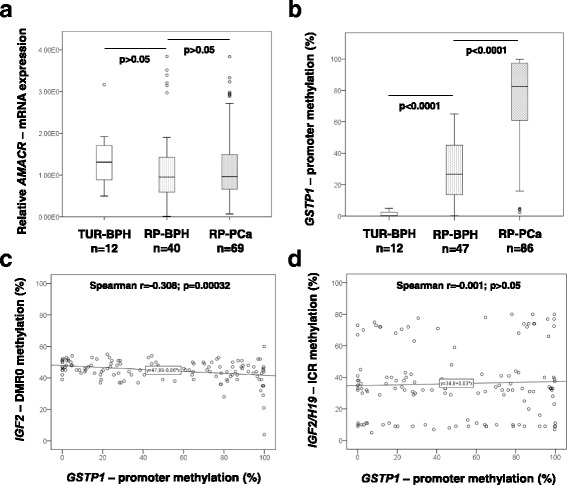
Studies on prominent biomarkers for prostate carcinogenesis AMACR and GSTP1. **a** Gene expression analyses of alpha-methylacyl-CoA racemase (AMACR) did not show significant differences between TUR-BPH, RP-BPH and RP-PCa; **b** A significantly increased *GSTP1* promoter methylation was observed by quantitative methylation specific PCR in RP-BPH and RP-PCa in comparison to TUR-BPH; **c** The Spearman’s correlation test considering all prostate tissue samples revealed a highly significant negative correlation between *GSTP1* promoter methylation and *IGF2*-DMR0 methylation; **d** Methylation levels in *IGF2/H19*-ICR and in *GSTP1* promoter were not correlated. Spearman’s rank correlation coefficients r and *p*-values are indicated

Two previously described tissue markers for prostate cancer, AMACR (alpha-methylacyl-CoA racemase) [46] and GSTP1 (Glutathione S-transferase P1) [47] were analyzed regarding the gene expression (*AMACR*) and promoter methylation (*GSTP1*), respectively, in tissue samples from TUR-BPH, RP-BPH and RP-PCa in order to additionally characterize the used tissue material. As expected, several RP-BPH and RP-PCa samples exhibited very high levels of *AMACR* gene expression, but, however, the differences regarding the median expression levels of *AMACR* were statistically not significant among the analyzed groups (Fig. 4a). In accordance to previous studies, a highly significant increase of *GSTP1* promoter methylation was detected in RP-BPH (median 26.6%, range 0.2–65%) and RP-PCa (median 82.5%, range 2.3–99.9%) in comparison to TUR-BPH (median 0.1%, range 0.04–4.9%) (TUR-BPH vs. RP-BPH: *p* < 0.0001; RP-BPH vs. RP-PCa: *p* < 0.0001, Mann-Whitney U test) (Fig. 4b). A significant negative correlation between *GSTP1* promoter methylation and *IGF2*-DMR0 methylation was found when considering all prostate cancer tissues (*p* = 0.00032, *r* = −0.308, Spearman’s rank correlation test) (Fig. 4c). The methylation in *GSTP1* promoter was not correlated to the methylation in *IGF2/H19*-ICR (Fig. 4d).

### The majority of prostate tumors exhibit unaltered copy numbers of IGF2 gene

**Fig. 5:**
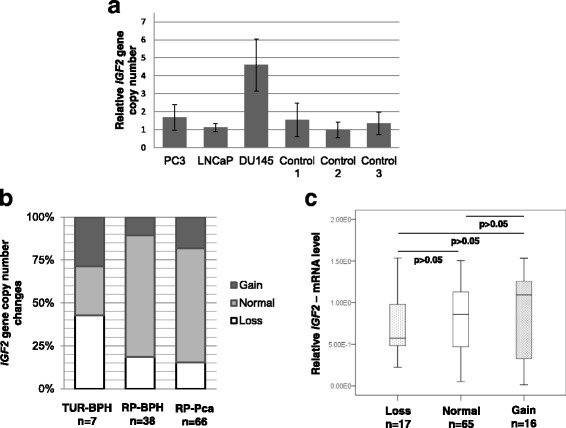
Analysis of *IGF2* gene copy number variation in prostate tumors and association to *IGF2*-mRNA expression. **a** Prostate cancer cell lines PC3, LNCaP and DU145 were analyzed together with three blood DNA samples (controls 1–3) regarding the copy number variation (CNV) of *IGF2* using the qBiomarker CNV PCR Assay (Qiagen). Increased *IGF2* copy numbers were found in DU145 cells, whereas in PC3 and LNCaP the values were comparable to controls. **b** *IGF2* CNV (gain, loss or normal) were analyzed in TUR-BPH (*n* = 7), RP-BPH (*n* = 38) and RP-PCa (*n* = 66). The majority (70% and 65%, respectively) of analyzed RP-BPH and RP-PCa samples showed normal copy numbers. **c** Tissue samples showing decreased, gained or normal copy numbers of *IGF2* were grouped and compared regarding *IGF2*-mRNA expression. No significant differences were found, although by trend, samples having increased *IGF2* copies showed higher median *IGF2*-mRNA values as those with decreased copies

In the course of carcinogenesis the gain or loss of *IGF2* gene copy numbers could be a reason for alterations in gene expression. In order to analyze, if the detected significant reduction of *IGF2* expression in RP-BPH and RP-PCa (Fig. 2a) is due to *IGF2* copy number changes, we applied a copy number variation (CNV) test. Prostate cancer cell lines PC3, LNCaP and DU145 were analyzed together with three blood DNA samples from healthy donors (controls 1–3) regarding the *IGF2* copy numbers using the qBiomarker CNV PCR Assay for Human chromosome 11 tile 10,752 (Qiagen) (Fig. 5a). Among the cell lines, DU145 cells showed a 4-fold increase of *IGF2* copies, whereas PC3 and LNCaP showed values similar to those in control samples (Fig. 5a). The *IGF2* CNV (gain, loss or normal) was then analyzed in primary prostate cancer tissue samples (7 TUR-BPHs, 38 RP-BPHs and 66 RP-PCas). The majority of RP-BPHs (71.1%) and RP-PCas (66.7%) exhibited unchanged copy numbers of *IGF2*. However, in TUR-BPH samples, 28.6% (*n* = 2) showed gain, 28.6% (*n* = 2) loss and 42.9% (*n* = 3) normal copy numbers of IGF2 (Fig. 5b). Samples having gain, loss or normal *IGF2* copy numbers were compared with *IGF2* expression. By trend, the median expression values for samples with a gain of *IGF2* copies were higher than for those with normal or decreased copy numbers (Fig. 5c). However, the differences were not significant (*p* > 0.05, Mann-Whitney U test). Moreover, the samples having normal and gained copy numbers also showed often substantially decreased *IGF2* expression values (Fig. 5c).

### Prostate cancer cell lines LNCaP and DU145 are applicable cell models for epigenetic studies on IGF2-DMR0 and its interaction with KLF4

**Fig. 6:**
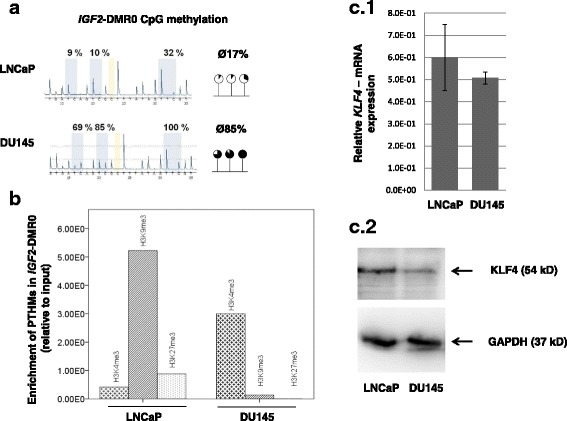
Epigenetic status of *IGF2*-DMR0 and expression of KLF4 in prostate cancer cell lines LNCaP and DU145. **a** Pyrograms show that LNCaP cells are hypomethylated in *IGF2*-DMR0 (average 17%), whereas DU145 cells are hypermethylated (average 85%); **b** Analysis of post-translational histone modification (PTHMs) by chromatin immunoprecipitation revealed an enrichment of H3K9me3 and H3K27me3 and a depletion of H3K4me3 in *IGF2*-DMR0 of LNCaP cells. In contrast, in DU145 cells an enrichment of H3K4me3 and a depletion of H3K9me and H3K27me3 was found; **c** Expression of KLF4 was confirmed in LNCaP and DU145 at mRNA level by RT-qPCR (**c.1**) and at protein level by western blot (**c.2**, GAPDH was used as control protein)

In order to analyze the binding of KLF4 in *IGF2*-DMR0 as a function of the methylation status, we analyzed two prostate cancer cell lines LNCaP and DU145, which exhibited an opposite methylation status in *IGF2*-DMR0 (LNCaP: average 17% methylation; DU145: 85%) (Fig. 6a) and expressed the transcription factor KLF4 at mRNA and protein levels (Fig. 6c1 and 2, respectively). For a detailed epigenetic characterization, both cell lines LNCaP and DU145 were then analyzed with regard to post-translational histone modifications (PTHMs) in *IGF2*-DMR0 (suppressive inactivating marks: H3K9me3 and H3K27me3; active transcription mark: H3K4me3). The hypomethylated *IGF2*-DMR0 in LNCaP cells (average methylation 17%) showed an enrichment of K3K9me3 and H3K27me3, and a depletion of H3K4me3. In contrast, the hypermethylated *IGF2*-DMR0 in DU145 cells (average methylation 85%) showed an enrichment of H3K4me3 and a depletion of H3K9me3 and H3K27me3 (Fig. 6b).

### KLF4 has a high potential to bind in the hypomethylated IGF2-DMR0 and to affect the IGF2 expression

**Fig. 7:**
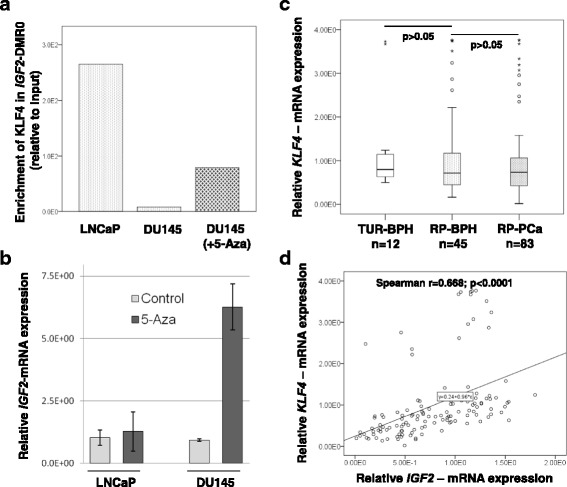
Enrichment of KLF4 in *IGF2*-DMR0 depends on *IGF2*-DMR0 methylation and affects the *IGF2*-mRNA expression. **a** Chromatin immunoprcipitation analyses revealed a high enrichment of KLF4 in *IGF2*-DMR0 in LNCaP cells (17% methylated in *IGF2*-DMR0) and low enrichment in DU145 cells (85% methylated in *IGF2*-DMR0). Treatment of DU145 cells with 5-Aza (DU145 + 5-aza) led to an increase of KLF4 binding in *IGF2*-DMR0. **b** Untreated (control) prostate cancer cell lines LNCaP and DU145 showed similar low levels of *IGF2* expression. Treatment with DNA methyltransferase inhibitor 5-aza-2′-deoxycytidin (5-Aza) caused a strong increase of *IGF2*-mRNA expression in DU145 cells, whereas LNCaP cells were unaffected. **c** Prostate tissue samples from TUR-BPH, RP-BPH and RP-PCa were analyzed regarding the *KLF4*-mRNA expression. No significant differences were found between the groups. **d** A highly significant positive correlation between *KLF4* and *IGF2* expression (Spearman’s rank correlation coefficient *r* = 0.668, *p* < 0.0001) was found, when all analyzed primary tumors were considered together without splitting in separate groups

The binding of KLF4 in the putative KLF4 consensus sequence was analyzed by ChIP in LNCaP and DU145 cells exhibiting different methylation states of *IGF2*-DMR0. A high enrichment of KLF4 was found in LNCaP cells with a hypomethylated *IGF2*-DMR0 (17%), whereas in DU145 cells with a hypermethylated *IGF2*-DMR0 (85%) KLF4 was absent (Fig. 7a). The cell lines were then treated with a demethylating substance 5-aza-2’deoxycytidin (5-aza, inhibitor of DNMT1) for 72 h. The treatment with 5-aza led to a binding of KLF4 in *IGF2*-DMR0 in DU145 cells (Fig. 7a). Moreover, the DNA demethylation by 5-aza led to a considerably increase of *IGF2*-mRNA expression in DU145 cells, whereas in LNCaP cells the *IGF2* expression remained at low levels comparable to those detected before 5-aza treatment (Fig. 7b).

### Expression of IGF2 gene in prostate cancer is significantly correlated with KLF4 expression

By comparing primary prostate cancer tissue samples grouped in TUR-BPH, RP-BPH and RP-PCa with regard to *KLF4* expression, we could not detect obvious differences (Fig. 7c). Tissue samples obtained by radical prostatectomy showed slightly decreased *KLF4* expression in comparison to samples obtained by transurethral resection of the prostate and comprised several samples with extreme high levels of *KLF4* (Fig. 7c). Interestingly, by considering all prostate tumors together without a separation in TUR-BPH, RP-BPH and RP-PCa, we found a highly significant correlation between *KLF4* and *IGF2* expression (*p* < 0.0001, Spearman’s rank correlation coefficient *r* = 0.668) (Fig. 7d).

## Discussion

Expression of the imprinted gene *IGF2* is often dysregulated in human cancer and the molecular mechanisms are still not fully understood. Our current study addressed potential mechanisms leading to transcriptional dysregulation of *IGF2* in prostate carcinogenesis. In this context we analyzed *IGF2* expression in prostate tissue obtained by radical prostatectomy (RP-PCa and adjacent RP-BPH) and transurethral prostate resection (TUR-BPH), and correlated it to different cancer associated processes as follows: 1. loss of imprinting (LOI) in *IGF;* 2. DNA methylation changes in the differential methylated region comprising promoter P0 of *IGF2* gene (*IGF2*-DMR0) and in the *IGF2/H19* imprinting control region located between *IGF2* and *H19;* 3. Gene copy number changes of *IGF2*. We have also observed the expression of *AMACR* and the methylation status of *GSTP1* promoter - both proposed tissue markers for prostate cancer. Taking into consideration the experiments performed in prostate cancer cell lines we reveal that hypomethylation of *IGF2*-DMR0 is a crucial point in deregulation of *IGF2* in prostate cancer and that the transcription factor KLF4 is a potent co-factor involved in impairment of *IGF2* expression during prostate carcinogenesis.

Loss of imprinting (LOI) in *IGF2* and an aberrant *IGF2* expression have been reported in human tumors of bladder, esophagus, colon, breast and prostate [16, 20, 23, 42]. It has been demonstrated that in case of PCa, the tumor-distant and -adjacent tissue samples possess higher IGF2 protein expression than the tumor itself and display also *IGF2* LOI [20]. This condition, described for PCa-adjacent and distant tumor-free prostate tissues, has been proposed as a pre-stressing event leading to tumorigenic transformation in long term [20]. Our data confirm the previous observations in prostate cancer and show additionally that not only BPH samples adjacent to PCa possess significantly higher *IGF2* expression as PCa itself, but also BPH obtained by transurethral prostate resection, i.e. benign prostatic hyperplasia without cancerous cells in the proximities.

Several studies on mouse models as well as human carcinomas demonstrated that altered high levels of IGF2 protein alone are not sufficient to trigger a tumorigenic transformation and that rather *IGF2* LOI, a proposed constitutive risk biomarker for colorectal cancer [12, 13, 44, 45], and *IGF2*-DMR0 hypomethylation seem to be indicative for tumor susceptibility [15, 16, 48]. In our study, most of the cases exhibiting *IGF2* LOI (8 out of 9 evaluated LOI) were detected in PCa and in PCa-adjacent BPH. The majority of heterozygous prostate cancer tissue samples, suitable for LOI studies (29 out of 38), exhibited maintenance of *IGF2* imprinting. We found that *IGF2* LOI in prostate cancer is not associated with methylation status in *IGF2*-DMR0, but associates significantly to hypermethylation of the imprinting control region (*IGF2/H19*-ICR) located between *IGF2* and *H19* genes.

In terms of *IGF2*-DMR0, it is known that this locus represents one of the two parentally imprinted regions within the *IGF2* gene (DMR0 and DMR2 in exon 9). *IGF2*-DMR0 possessing promoter activities, is normally hypermethylated at the active paternal allele and was shown to acquire somatically a hypomethylated state in human cancer [19, 22, 23, 41, 42]. In accordance with other publications, we observed a hypomethylation of *IGF2*-DMR0 in PCa and in PCa-adjacent BPH, which was significantly different to non-cancerous BPH obtained by transurethral resection. In contrast, methylation in *IGF2/H19*-ICR was not significantly changed in the course of prostate carcinogenesis.

In carcinogenesis, the gain or loss of *IGF2* gene copy numbers (copy number variation, CNV) could be a reason for aberrant gene expression levels. In order to address this issue we examined *IGF2* mRNA levels in prostate tissue samples exhibiting gain, loss and normal copy numbers of *IGF2*. The samples having gained *IGF2* copies exhibited the highest median values for *IGF2* mRNA, and those with a loss – the lowest. The differences between the groups were not statistically significant, and moreover, several tumor samples with increased copy numbers exhibited low *IGF2* mRNA and, vice versa, several samples with decreased copy numbers possessed high levels of *IGF2* mRNA. Thus, CNV cannot per se explain aberrant *IGF2* expression in prostate tumors.

Further, we observed a significant inverse correlation between *IGF2*-DMR0 and *GSTP1* promoter methylation (*r* = −308, *p* = 0.00032). Hypermethylation of *GSTP1* promoter is a hallmark of prostate carcinoma [47] and high methylation rates were associated with more aggressive tumor stages with a Gleason score ≥ 4 + 3 [49]. The fact that *IGF2*-DMR0 hypomethylation goes along with *GSTP1* hypermethylation suggests that *IGF2*-DMR0 hypomethylation could be also considered for risk assessments of prostate cancer. However, *GSTP1* methylation was not correlated to methylation in *IGF2/H19*-ICR.

In order to determine potential co-factors for regulation of *IGF2* expression, we analyzed the *IGF2*-DMR0 region with regard to putative binding sites for transcription factors and revealed a consensus motif for KLF4. The transcription factor KLF4 belongs to a subgroup of zinc finger proteins and was shown to bind to G/C-rich DNA. Interestingly, hypomethylation of three specific CpGs within the *IGF2*-DMR0, two of which belong to our identified and analyzed KLF4 consensus sequence, was shown to be closely linked to *IGF2* LOI in human tumorigenic tissues of breast, colon and esophagus [23, 42]. Our study revealed that KLF4 binds to *IGF2*-DMR0 in dependence to the prevailing epigenetic status and affects *IGF2* gene expression. Utilizing prostate cancer cell lines, we found in LNCaP that KLF4 binds to hypomethylated *IGF2*-DMR0 and co-localizes with H3K27me3 and H3K9me3. No binding was detectable in DU145, when *IGF2*-DMR0 was hypermethylated and enriched with H3K4me3. Treatment of DU145 cells with the demethylating substance 5-aza-2-‘deoxycytidin led to a 4-fold increase of *IGF2* expression. A recent study demonstrated that repression of the maternal *IGF2* allele, which is normally unmethylated, is achieved by binding of PRC2 (polycomb repressive complex) components H3K27me3 and H3K9me3 to *IGF2*-DMR0 [50, 51]. Our results suggest that KLF4 could be involved in repression of the maternal *IGF2* allele. The epigenetic imbalance in *IGF2*-DMR0 and the ability of KLF4 to bind here and to affect *IGF2* expression should be investigated in further studies in more detail with regard to other PRC2 components and to functional consequences.

In terms of prostate carcinogenesis, inhibition of the KLF4/P13/Akt/p21 pathway by microRNA-7 repressed the stem cell attributes of PCa cells and their tumorigenic potential [52]. Furthermore, KLF4 has been reported to mediate lysophosphatidic-acid stimulated migration and proliferation of PC3 cells [53] and to be associated with proliferative activity of PCa cells through the KLF4-KRT6/13 pathway [38]. However, an earlier study in prostate cancer cell lines, where a RNA- and vector-mediated KLF4 over-expression was achieved, suggested that KLF4 can also act in a tumor suppressive manner [54]. In primary prostate tumors, examinations on tissue samples from age matched patients by immunohistochemistry showed that KLF4 protein is expressed in the vast majority of epithelial cells in BPH as well as in PCa, whereby PCa exhibited lower KLF4 expression as BPH [37]. Non-tumorous areas of the prostate exhbited at both, mRNA and protein levels, a KLF4 expression similar to BPH [36, 55]. Our analyses in prostate tissue samples revealed at the mRNA level that *KLF4* expression is slightly decreased in RP-PCa in comparison to RP-BPH and TUR-BPH. However, the differences were not statistically significant, and some samples, particularly in RP-PCa and RP-BPH group, possessed also very high levels of *KLF4* expression. What is striking is the fact that, when considering all tumor samples together without separation in groups, we find a highly significant correlation of *KLF4* and *IGF2* expression (*p* < 0.0001, Spearman’s rank correlation coefficient *r* = 0.668). The latter indicates that within a same prostate tumor tissue the *IGF2* expression is interconnected to the *KLF4* expression.

## Conclusions

In conclusion, our analyses reveal a potent role of KLF4 transcription regulation on *IGF2* in prostate cancer. We demonstrate that, in addition to the DNA methylation degree, the prevailing histone-modifications in *IGF2*-DMR0 are critical for KLF4 binding and *IGF2* up- or down-regulation, respectively. Our clinical studies show that the majority of PCa and adjacent BPH, but not BPH obtained by TUR, possess severely reduced *IGF2* expression and hypomethylated *IGF2*-DMR0. Expression levels of *IGF2* and *KLF4* were highly correlated in the different prostate tissues. Moreover, we reveal a significant correlation of *GSTP1* hypermethylation and *IGF2*-DMR0 hypomethylation suggesting the latter as a conceivable biomarker for prostate carcinogenesis. *IGF2* LOI and CNV occurring in the course of prostate tumorigenesis seem not to be the decisive factors for aberrant *IGF2* expression. Collectively, our study provides novel insights into *IGF2* deregulation mechanisms as a critical process in prostate tumorigenesis.

## Abbreviations

AMACR: Alpha-methylacyl-CoA racemase
BPH: Benign prostate hyperplasia
ChIP: Chromatin-immunoprecipitation
CNV: Copy number variation
CTCF: CCCTC-binding factor
GAPDH: Glyceraldehyde 3-phosphate dehydrogenase
GSTP1: Glutathion S-Transferase P1
IGF2: Insulin-like growth factor 2
IGF2/H19-ICR: Imprinting control region regulating parental allele expression of IGF2 and H19 genes
IGF2-DMR0: Differential methylated region in promoter 0 of the IGF2 gene
KLF4: Kruppel-like factor 4
LOI: Loss of imprinting
MOI: maintenance of imprinting
PCa: Prostate carcinoma
PTHM: Post-translational histone modification
RP-BPH: BPH obtained by radical prostatectomy
RP-PCa: PCa obtained by radical prostatectomy
RT-qPCR: Reverse transcription followed by quantitative polymerase chain reaction
TUR-BPH: BPH obtained by transurethral resection of the prostate
